# 

**Published:** 1892-01

**Authors:** 


### INDEX

TO THE

### HOMŒOPATHIC PHYSICIAN.

### VOLUME XII, 1892.

PAGE

A, B, C of the Swedish System of Educational Gymnastics. By Hartvig Nissen. Review of, . . . . . . 121
Abscesses of Vulva Cured by Sepia. I. Dever, M. D., . . . . . . . . . 548
Abstract of the Symptons, An. Reed & Carnrick, Review of, . . . . . 77
Aconite, . . . . . . . . . . . . . . . . . 84
Additional Report of the Board of Censors, . . . . . . . . . . . . 498
Adeps, . . . . . . . . . . . . . . . . 524
Agaricus-musc., . . . . . . . . . . . . 524
Age of the Domestic Animals. By Rush Shippen Huidekoper, M. D. Review of, . . . . . . . . . . 76
A Good Opening for a Physician. Note, 464
All Around the Year. Lee & Shepard's Calendar, Notice of, . . . 78, 584
Allen, Arthur G., M. D. *Organon*, § 16, 430
Allen, J. H., M. D. Homœopathy a True Science, . . . . . . . . . . 418
Verifications of Medorrhinum, . . 531
[illegible] Association of the Hahnemann Medical College, The. W. [illegible] Van Baum, M. D., . . . . . . 173
American Anti-Vaccination League, The. Note, . . . . . . . . . . . 79
American Institute of Homœopathy, The . . . . . . . . . . . . 161, 321, 322
American Obstetrical Society. Notice, . . . . . . . . . . . . . . . 162
American Text-Book of the Theory and Practice of Medicine, The. Note, . . . . . . . . . . . . . . 80
Ammon-mur., . . . . . . . . . . . . . 84
Analytical Symptomatology of the Homœopathic Materia Medica. By Rufns L. Thurston, M. D., and Samuel A. Kimball, M. D. Review of, . . . . . . . . . . . 367
Annual Report of the Postmaster-General of U. S. for 1891. Review of, . . . . . . . . . . . . . . . . 76
Antirrhinum Linaria, . . . . . . . . 55
Apocynum-cann., . . . . . . . . . . . 524
Apology, An, . . . . . . . . . . . . . 272
Aranea-diad, . . . . . . . . . . . . . 524
Arsenic, . . . . . . . . . 29, 84, 523, 524, 526
Arsenic, A Symptom of. Ella M. Tuttle, M. D., . . . . . . . . . . . 29
Arum-triph, . . . . . . . . . . . . . 50
Asclepias-tub., . . . . . . . . . . . . 50
Association of Military Surgeons, The, 80
Asterias-rub., . . . . . . . . . . . . . 50
Atropine, . . . . . . . . . . . . . . 51
Atropine Sulphate, . . . . . . . . . . 51
Attenuation, The Giant of. Hayes C. French, M. D., . . . . . . . . . . 397
Aurum-mur., . . . . . . . . . . . . 51, 524
Aurum m. Nasal Polypi Cured with One Dose of. J. R. Haynes, M. D., . . . . . . . . . . . . . . . 551

Bacteriological Diagnosis. By James Eisenberg, M. D. Review of, . 267
Ballard, Edgar Abbott, M. D. In Memoriam. Hitchcock, . . . . . 6
Baptisia-tinc., . . . . . . . . . . . . 277
Baruch, Dr. Meyer. Note, . . . . . . 79
Baryta-carb., . . . . . . . . . . . . 277
Bayard, Edward, M. D. The Vital Force Defined, . . . . . . . . . 63

PAGE

Beaman, Dr. Charles P. Note, . . . . 316
Belladonna, . . . . . . . . . . . 84, 277, 524
Bender, Dr., has removed. Notice of, . . . . . . . . . . . . . . 528
Bentley, W. R., M.D. Is the Indicated Remedy an All-Sufficient? . . . 303
Benzoic Acid, . . . . . . . . . . . . 278
Berberis, . . . . . . . . . . . . . . . 84
Blanke, Dr. T. F., has removed. Note, 528
Boger, C. M., M. D. Perverted Appetite and Impotence, . . . . . . 116
Book Notices, . . 32, 76, 121, 174, 220, 266, 313, 365, 408, 463
Borax, . . . . . . . . . . . . . . . . 278
Boston University School of Medicine, . . . . . . . . . . . . . . 78
British Medicinal Plants. Alfred Heath, M. D., . . . . 16, 65, 168, 258
Broken Doses and the Repetition of the Dose. Walter M. James, M. D., . . . . . . . . . . . . . . 417
Bromine, . . . . . . . . . . . . . . 278
Bryonia-alb., . . . . . . . . . . . . 84, 279
Bufo, . . . . . . . . . . . . . . . . 279
Bureau of Materia Medica, . . . . . . 499
Bureau of Service and Information, A. Note, . . . . . . . . . . . 318
Bursa-pastoris, . . . . . . . . . . 279, 524

Cactus-grand, . . . . . . . . . . . . 284
Cadmium-sulph., . . . . . . . . . . . 282
Calc-carb., . . . . . . . . . . . . . . 281
Calcarea-Ostrearum, A Study. A. McNeil, M. D., . . . . . . . . . . 100
Calcium Salts in Nutrition, The. Note, . . . . . . . . . . . . . . 318
Calendula-off., . . . . . . . . . . . . 280
Camphor, . . . . . . . . . . . . . 283, 286
Cancer-fluv., . . . . . . . . . . . . . 280
Cancer-astac., . . . . . . . . . . . . 524
Cantharis. What did it Cure? Frank Kraft, M. D., . . . . . . . . . . 157
Can there be Drug Action without Drug Presence? . . . . . . . . 24
Canton Surgical Chair Co., The. Note, 272
Capsicum, . . . . . . . . . . . . . 84, 280
Carb-veg., . . . . . . . . . . . . . . 285
Carbolic-acid, . . . . . . . . . . . . 281
Carb-sulph., . . . . . . . . . . . . . 281
Case, E. E., M. D. Some Characteristics of Kali-phosphoricum, . . 144
Three Surgical Cases, . . . . . . . 543
Cases from Practice. Dr. Hesse, . . . 406
Case Cured. Alexander Villers, M. D., 403
Caulophyllum-thal., . . . . . . . . . 281
Causticum, . . . . . . . . . . . . . . 285
Cenchris Contortrix. Wm. Steinrauf, M. D., . . . . . . . . . . . . . . 173
Censors, Additional Report of Board of, . . . . . . . . . . . . . . . . 498
Cereus Bonplandii. J. H. Fitch, M. D., . . . . . . . . . . . . . . . . 533
Chamomilla, . . . . . . . . . . . . 84, 281
Chelidonium-majus, . . . . . . . . . 280
Chelon-glab., . . . . . . . . . . . . 281
Childhood. By Dr. Geo. W. Winterburn and Miss Florence Hall. Review of, . . . . . . . . . . . 365, 528
China-off, . . . . . . . . . . . . . . 282
Chinese, The; Their Present and Future. By Robert Coltman, Jr., M. D. Review of, . . . . . . . 174

PAGE
Chloral-hydrat, . . . . . . . . . . . . . 282
Choate, Rufus, M. D. The Beneficence of Homœopathy, . . . . 311
Homœopathic Dilutions, . . . . . 68
A Lac-Caninum Case, . . . . . . 166
Chronic Disease Forces or Miasms. Ed. Cranch, M. D., . . . . . . . 130
Cicatricial Tissue: Graphites *vs.*; Caroline E. Hastings, M. D., . . 553
Cichorium-intybus, . . . . . . . . 280, 282
Cimicifuga-rac., . . . . . . . . 282, 285, 524
Cina, . . . . . . . . . . . . . . . . . 285
Cina. Thomas Wildes, M. D., . . . . 156
Circumcision, History of, from the Earliest Times to the Present. By P. C. Remondino, M. D. Review of, . . . . . . . . . . . . 175
Clark, Dr. George H. Has removed. Note, . . . . . . . . . . . . . . 32
Medorrhinum, . . . . . . . . . . . 499
Some Symptoms of Sycosis, . . . 451
Clarke, Dr. John H. Removal. Note, 78
Climatologist, The. By John M. Keating, M. D. Review of, . . . . . 269
Clinical Cases. W. E. Ledyard, M. D., . . . . . . . . . . . . . . . 10
Clinical Experience, Some. J. H. Jackson, M. D., . . . . . . . . . 159
Clinical Notes. I. N. R., . . . . . . . 92
Clinical Text-Book of Medical Diagnosis for Physicians and Students. By Oswald Vierordt, M.D. Review of, . . . . . . . . . . . 221
Cleary, Mr. W. P. Note, . . . . . . . 272
Clematis-virg., . . . . . . . . . . . . . 282
Cleveland Homœopathic Medical College, The. Note, . . . . . . . . 223
Close, Stuart, M. D. Truth and Error in Vaccination, . . . . . . . . 468
Cobalt-chloride, . . . . . . . . . . . 285
Cochlearia-arm., . . . . . . . . . . 286, 525
Coffea-cruda, . . . . . . . . . . . . 84, 282
Coffea-cruda in Enuresis. J. C. White, M. D., . . . . . . . . . . . . . . 14
Cold, Sensations of, . . . . . . . . . 143
Collinsonia-can., . . . . . . . . . . . 232
Colocynth, . . . . . . . . . . . . . . . 84
Conium-maculatum, . . . . . . . . . 283
Copaiba, . . . . . . . . . . . . . . . . 286
Commentaries upon *The Organon*. B. Fincke, M. D., . . . . . . . . 246, 374
Committee of the American Public Health Association, The. Note, 128
Concussion of the Brain. Sarah N. Smith, M. D., . . . . . . . . . 400
Consumption: How to Prevent it and How to Live with it. By Dr. U. S. Davis, M. D. Review of, . 122
Consumption, The New Cure of, by its Own Virus. By J. Compton Burnett, M. D. Review of, . . 124
Copper, An Experience with. L. M. Stanton, M. D., . . . . . . . . 249
Cornus-florida, . . . . . . . . . . . 283, 525
Cranch, E., M. D. Chronic Disease Forces, or Miasms, . . . . . . . 130
Cranch, Edward, M. D. Use of Forceps, . . . . . . . . . . . . . . . 164
Cresotum, . . . . . . . . . . . . . . . 283
Croton-tiglium, . . . . . . . . . . . 280
Cupri-acetas, . . . . . . . . . . . . . 286
Cuprum-acet., . . . . . . . . . . . 284, 525
Cuprum-met., . . . . . . . . . . . . . 284

Dangerous Proximity. Note, . . . . 416
Death in the Communion Cup. Note, 80
Definition of Apothecary. Note, . . 416
Delayed August Number, The. Note, 322
Delivery by Forceps and Subsequent Anasarca. John Hall, M. D., . 112
Dever, I., M. D. Abscesses of Vulva Cured with Sepia, . . . . . . . 548
Facts, not Theories, . . . . . . . 19
Thoughts on the Psoric Miasm, . 456

PAGE
Dillingham, Thos. M., M. D. Infantile Spinal Atrophic Paralysis and other Cases, . . . . . . . . . . . 564
Discussion of Dr. McLaren's Paper at the Meeting of the I. H. A., June 24th, 1891, . . . . . . . . . 110
Did not Earn His Fee. Note, . . . . 128
Digitalis Purpurea, . . . . . . . . . 286, 525
Dios Chemical Co., The. Note, . . . . 317
Diphtheria, A Case. L. Hoopes, M. D., 364
Diseases of the Eye. By G. E. de Schweinitz, M. D. Review of, 314
Doesn't Cost Anything. Note, . . . . 128
Dolichos Pruriens, . . . . . . . . . . 287
Doryphora-decemlineata, . . . . . 287
Dracontium Fœtidum, . . . . . . . . 286
Drake, Dr. Olin. Note, . . . . . . 124, 316
Drawing the Line. Hayes C. French, 313
Drevet Manufacturing Co. Note, . . 79
Drosera, . . . . . . . . . . . . . . . . 84
Drug Action without Drug Presence, Can There be, . . . . . . . . . 24
Dudley Pemberton, M. D. American Institute of Homœopathy, . . . 161
Note, . . . . . . . . . . . . . . . . 414

Editorials, . . 1, 33, 81, 129, 177, 225, 273, 321, 369, 417, 465, 529
Electric Review, The. Notice of, . . 464
Electro-Homœopathy. Note, . . . . 272
Emmons, J., M. D. A Few Cases Cured with the High and Highest Potencies, . . . . . . . . . 364
End of the Year, The, . . . . . . . . 529
Epilepsy. D. C. McLaren, M. D., . . 108
Epilepsy, Dr. McLaren's Paper in March Number. Hattie C. Van Buren, M. D., . . . . . . . . . . 265
Erecthites-hieracefolia, . . . . 288, 522, 525
Erigerion-can., . . . . . . . . . . . . 288
Error in the Lippe Repertory, An. S. Mills Fowler, M. D., . . . . . . 31
Essentials of Medical Electricity. By D. D. Stewart, M. D. Review of, . . . . . . . . . . . . . . . 122
Essentials of Physics. By Fred. J. Brockway, M. D. Review of, . . 122
Ether-sulphuric, . . . . . . . . . . . 288
Eupatorum-perfoliatum, . . . 287, 288, 525
Euonymus-atrop., . . . . . . . . . . 288
Eupion, . . . . . . . . . . . . . . . . 288

Facts, Not Theories. I. Dever, M. D., 19
Felix-mas., . . . . . . . . . . . . . . 289
Femur, Fracture of the—An Appeal. T. Dwight Stow, M. D. . . . . . 297
Ferri-carb., . . . . . . . . . . . . . . 289
Ferrum-met., . . . . . . . . . . . . 84, 288
Fincke, B., M. D. Commentaries upon *The Organon*, . . . . 179, 246, 374
In Memoriam, P. P. Wells, M. D., 9
Neural Analysis, . . . . . . . . . . 424
First Decennial Catalogue of the College of Physicians and Surgeons of Chicago. Notice of, . . . . . 313
Fitch, J. H., M. D. Cereus Bonplandii, . . . . . . . . . . . . . . . 533
Fluoric-acid, . . . . . . . . . . . . . 289
Forceps, Use of. Edward Cranch, M. D., . . . . . . . . . . . . . . 164
Formica-rufa, . . . . . . . . . . . 288, 525
Fowler, S. Mills, M. D. An Error in the Lippe Repertory, . . . . . . 31
Homœopathic Dilutions, . . . . . 69
Note, . . . . . . . . . . . . . . . . 317
Fragmentary Proving of Potassium Chlorate. E. Rushmore M. D., 530
French, Hayes C., M. D., Drawing the Line, . . . . . . . . . . . . . . 313
The Giant of Attenuation, . . . . 397
Frothingham, Professor A. L. Note, 317
Fun for Doctors, . . . . . . . . . 128, 416

Gelsemium-semper, . . . . . . . . . 290
Gettysburg Spring Water, . . . . . . 291

PAGE
Ginseng, . . . . . . . . . . . . . . . . 290
Givens. Dr. Amos. Has removed. Note, . . . . . . . . . . . . . . . 124
Glanderine, . . . . . . . . . . . . . . . 290
Globe Patent Agency, The. Note, . . 79
Graphalium, . . . . . . . . . . . . . . 84
Graphites, . . . . . . . . . . . . . . . . 291
Graphites *vs.* Cicatricial Tissue. Caroline E. Hastings, M. D., . . . . 553
Green, Mrs. M. J., M. D. Utah Homœopathic Medical Association, . . 309
Griffith, Dr. F. L. Note, . . . . . . . 414
Guaiacum, . . . . . . . . . . . . . . . 290
Guiding Symptoms of Our Materia Medica. By C. Hering, M. D. Review of, . . . . . . . . . . . . 124
Gummi-gutti, . . . . . . . . . . . . . 290
Gymnastics, Swedish System. Review of, . . . . . . . . . . . . . . . 121

Hahnemann Hospital, The. Note, . . 127
Hahnemannian Consultation Blank, The. S. Mills Fowler, M. D. Notice of, . . . . . . . . . . . . . 78
Hall, John, M. D. Delivery by Forceps and Subsequent Anasarca, 112
Hamamelis Virginiana. Some Remarks on. Dr. Bree. . . . . . . 61
Hamemelis-virg., . . . . . . . . . . . 291
Harvey, Dr. Walter E. Removal. Note, 78
Hastings, Caroline E., M. D. Graphites *vs.* Cicatricial Tissue, . . . 553
Haynes, J. R., M. D. Ipecac in Uterine Hemorrhage, . . . . . . . . 251
Is Similia a Universal Law? . . . 448
Nasal Polypi cured with One Dose of Aurum-m., . . . . . . . . . . 551
Hazard, Dr. T. L. Has removed. Notice of, . . . . . . . . . . . 528
Heath, Alfred, M. D. British Medicinal Plants, . . . . . . 16, 65, 168, 258
Hellebore, . . . . . . . . . . . . . . . 292
Hepar-sulph., . . . . . . . . . . . . . 292
Hering College of Homœopathy, The. 370, 414
Hermance, Geo. A., M. D. A Case of Traumatism, . . . . . . . . . . 107
Hernia. Wm. Steinrauf, M. D., . . . 91
Hesse, Dr. Cases from Practice, . . . 407
Cures by Lycopodium, . . . . . . 360
Highest Potencies. A Few Cases Cured with the High and Highest. J. Emmons, M. D., . . 364
Hitchcock. In Memoriam. Edgar Abbott Ballard, M. D., . . . . . 6
Hitchcock, Haslyn, M. D. Vaccination and Homœopathy, . . . . 476
Holmes, Horace P., M. D. A Case of Sick Headache, . . . . . . . . 37
Has removed. Note, . . . . . . . 32
The Use of Repertories in Finding the Homœopathic Remedy, . . 36
Homœopathic Bibliography of the United States. By Thomas Lindsley Bradford, M. D. Review of, 410
Homœopathic Dilutions. Rufus Choate, M. D., . . . . . . . . 68
Homœopathic Dilutions. S. Mills Fowler, M. D., . . . . . . . . . 67
Homœopathic Journal of Obstetrics, Gynecology, and Pedology, The. Review of, . . . . . . . . . 269, 367
Homœopathic Medical College of Missouri, The. Note, . . . . . 222
Homœopathic Medical Society of Ohio, The. Note, . . . . . . . . 223
Homœopathic Medical Society of Wisconsin, The. Note, . . . . 315
Homœopathic Therapeutics of Hemorrhoids. By Wm. Jefferson Guernsey, M. D. Review of, . . 271
Homœopathy, The Beneficence of. Rufus Choate, M.D., . . . . . . 311
Homœopathy in Hungary. Note, . . 319
Homœophy or Isopathy? Is it. S. Swan, M. D., . . . . . . . . . . 75
Homœopathy, Obstacles to. Note, . . 315
Homœopathy a True Science. J. H. Allen, M. D., . . . . . . . . . 418
Homœopathy in the United States. Note, . . . . . . . . . . . . 413
Hoopes, L., M. D. The Oldest Homœopathic Physician, . . . . . 31
Diphtheria—A Case, . . . . . . . . 364
Horlick's Malted Milk. Note, . . . . 368
Howard, Clarence C., M. D. Detachment of the Retina, . . . . . . 23
Hydrophobinum, . . . . . . . . . . . 292
Hyoscyamus, . . . . . . . . . . . . . . 292
Hypericum-perfol., . . . . . . . . . 291, 525

Ignatia, . . . . . . . . . . . . . . . . 85
Importance of Moral Symptoms, . . . 92
Important Notice and Removal. Note, 126, 272
Impotence and Perverted Appetite. C. M. Boger, M. D., . . . . . . . 116
In Convalescence. Note, . . . . . . 464
Indiana Institute of Homœopathy, . 393
Infant Feeding. I. H. A., . . . . . . 43
Infantile Spinal Atrophic Paralysis. Thomas M. Dillingham, M. D., . 564
Influence of Medicine upon Character, The. Walter M. James, M. D., . . . . . . . . . . . . . . 33
In Memoriam. Dr. Giles M. Pease, .
J. M. Selfridge, M. D., . . . . . . 260
Thomas F. Pomeroy, M. D., . . . 216
M. A. A. Wolff, M. D., . . . . . . 8
I. N. R. Clinical Notes, . . . . . . . 92
International Hahnemannian Association, 319, 322, 349, 418, 468, 495, 530
International Medical Annual and Practitioners' Index. Percy Wilde, M. D. Review of, . . . 267
Iodide of Potassium, . . . . . . . . . 51
Iodine, . . . . . . . . . . . . . . . 51, 293
Ipecac in Uterine Hemorrhages. J. R. Haynes, M. D., . . . . . . . 251
Ipecac, . . . . . . . . . . . . . . . . 51
Iris, . . . . . . . . . . . . . . . . . 85
Is the Indicated Remedy an All-Sufficient? W. R. Bentley, M. D., . 303

Jackson, J. H., M. D. Some Clinical Experience, . . . . . . . . . . 159
Jaeger, Prof. Dr. Gustav. Potentiation Physiologically Proven, 228, 328, 370
James, Walter M., M. D. The American Institute of Homœopathy, . 321
Broken Doses and the Repetition of the Dose, . . . . . . . . . . 417
The Doctrine of Reflexes . . . . . 273
The End of the Year, . . . . . . . 529
The First Paragraph of *The Organon*, . . . . . . . . . . . . . 81
The Influence of Medicine upon Character, . . . . . . . . . . . 33
The International Hahnemannian Association, . . . . . . . . 322
Not For Sale, . . . . . . . . . . . 3
The Portrait of Adolph Lippe, M. D., . . . . . . . . . . . . . . . 129
Salutatory, . . . . . . . . . . . . 1
Should the Patient be Told what Remedy He Is Taking? . . . . 225
Truth and Error in Vaccination, . 465
Jasper, . . . . . . . . . . . . . . . . 52
Jaspis, . . . . . . . . . . . . . . . . 526
Journals for Sale. Note, . . . . . . . 127

Kali-bichrom., . . . . . . . . . . . 52, 85
Kali-carb., . . . . . . . . . . . 52, 85, 525
Kali-chloricum, Fragmentary Proving of. E. Rushmore, M. D., . 530
Kali-hyd., . . . . . . . . . . . . . . 52, 85
Kali-phosphoricum, Some Characteristics of. E. E. Case, M. D., . . 144
Kaolin, . . . . . . . . . . . . . . . . 52

PAGE
Kennedy, A. L., M. D. Note, . . . . 414
Key Notes. Samuel Swan, M. D., . . 164
Klip, The. Note, . . . . . . . . . . . 125
Knew His Business. Note, . . . . . 416
Kraft, Frank, M. D. What Did Cantharis Cure? . . . . . . . . . . . 157
Kreosote, . . . . . . . . . . . . . . . 53, 85
Kunkel, Dr. Sycosis Menti, . . . . . 201

Lac-caninum Case, A. Rufus Choate, M. D., . . . . . . . . . . . . . . 166
Lac-can., . . . . . . . . . . . . . . . . 85
Laceration of the Cervix Uteri (?) Cured by Homœopathic Treatment. A. Quackenbush, M. D., 516
Lachesis, . . . . . . . . . . . . . 53, 85, 525
Lachesis and Sepia Cases. P. C. Sanderson, M. D., . . . . . . . . . 463
Lachesis, Sick Headache Cured with One Dose of. Edward Rushmore, M. D., . . . . . . . . . . . 64
Lactuca-viros, . . . . . . . . . . . . 54
Lamium, . . . . . . . . . . . . . . . 54
Laurocerasus, . . . . . . . . . . . . 54
Laurocerasus, Fearful Aggravation Caused by. Geo. Wigg, M. D., 30
Leach, Dr. R. B. Note, . . . . . . . 528
Ledum, . . . . . . . . . . . . . . . 54, 85
Ledyard, W. E., M. D. Clinical Cases, 10
Leslie, Martin, M. D. Spinal Congestion or Spinal Myelitis, . . . . . 155
Lies, A Refuge of. Note, . . . . . . . 79
Life Insurance Inquiry, . . . . . . . 125
Lilium-tigrinum, . . . . . . . . . . . 55
Linaria-vulg., . . . . . . . . . . . . 55, 526
Lippe, The Portrait of Adolph, M. D., 129
Lippe, The Portrait of Dr. Adolph. C. C. Smith, M. D., . . . . . . . . 215
Lippe's Repertory, Error in. S. Mills Fowler, M. D., . . . . . . . . . . 31
Lithia-carb., . . . . . . . . . . . . . 53, 54
Lobelia, . . . . . . . . . . . . . . . . 55
Lungs. A Case of Ulceratian of the. G. J. Waggoner, M. D., . . . . . 114
Lutze, Dr. F. H. Note, . . . . . . . . 317
Lycopodium, Cures by. Dr. Hesse, . 360
Lycopodium, . . . . . . . . . . . . . 55, 85

Macfarlan, Malcolm, M. D. Provings and Clinical Observations with High Potencies, . 50, 93, 134, 277, 522
Mack, Charles S., M. D. Swedenborg and Medicine, . . . . . . . . . . 118
Malarial Fever in the Sacramento Valley. James T. Martin, M.D., 239
Malted Milk, . . . . . . . . . . . . . 464
Martin, James T., M. D. Malarial Fever in the Sacramento Valley, 239
Martin, Leslie, M. D. A Case of Twin Pregnancy Simulating Fibroid Tumors, . . . . . . . . . . . . . 149
Materia Medica, A New. Note, . . 223, 367
McClelland, Dr. J. H. Note, . . . . . 412
McLaren, D. C., M. D. Epilepsy, . . 108
McNeil, A., M. D. Calcarea-Ostrearum. A Study, . . . . . . . . . 100
Medical Monopoly, . . . . . . . . . . 405
Medical Treatment of Sick Ants. Note, . . . . . . . . . . . . . . . 126
Medical Visitor, The. Note, . . . . . 319
Mediterranean Shores of America. By P. C. Remondino, M. D. Review of, . . . . . . . . . . . . . . . 221
Medorrhinum, Verifications of. J. H. Allen, M. D., . . . . . . . . . . . 531
Medorrhinum. Geo. H. Clark, M. D. 499
Thomas Wildes, M. D., . . . . . . 70
Mentha-pip., . . . . . . . . . . . . . 56
Mephitis, . . . . . . . . . . . . . . . 56
Metastasia, A Case of. Wm. Steinrauf, M. D., . . . . . . . . . . . . . . 172
Mercurius corrosivus, . . . . . . . 56, 527
Merc-prot-iod., . . . . . . . . . . . 57, 526
Merc-viv., . . . . . . . . . . . 57, 526, 527
Mezereum, . . . . . . . . . . . . . . 58
Missouri Institute of Hom, The. Note, 126
Morgan, A. R., M.D. Some Thoughts upon Potentization and Dose, . 383
Morphia Habit, The, . . . . . . . . . 119
Muriatic Acid, . . . . . . . . . . . 58, 523
Myrica Cerifera, . . . . . . . . . . . 58
Myrtus corn., . . . . . . . . . . . . . 58

Natrum-mur., . . . . . . . . . . . . 58
Natrum-sulph., . . . . . . . . . . . . 85
Necrosis of Os Calcis. G. W. Sherbino, M. D., . . . . . . . . . . . 555
Neural Analysis. B. Fincke, M. D., . 424
Neural Analysis of the Potencies of Kali-carbonicum, . . . . . . . . . 330
New Pronouncing Dictionary of Medicine. By John M. Keating, M. D. Review of, . . . . . . . . . 411
New York Homœopathic Union. L. M. Stanton, M. D., . . . . . . . 3, 255
Nitric Acid, . . . . . . . . . . . . 59, 526
Nitrum, . . . . . . . . . . . . . . . 59
Northern Indiana and Southern Michigan Homœopathic Medical Association. Note, . . . . . . . . . 316
Nosode Cases. J. T. Tapley, . . . . . 520
Notes from Past Meetings of the Lippe Society, . . . . . . . . . . . . . 467
Notes and Notices . . . . 32, 78, 124, 222, 271, 315, 368, 412, 464, 528
Nux-moschata, . . . . . . . . . . . . 59
Nux-vomica, . . . . . . . . . . . . 59, 85

Oleander, . . . . . . . . . . . . . . 60
O'Connor, Dr. Joseph T., has Removed. Note, . . . . . . . . . . 528
Oldest Homœopathic Physician, The. L. Hoopes, M. D., . . . . . . . . . 31
Oldest Homœopathic Physician, The. L. B. Wells, M. D., . . . . . . . . 113
Only Body Snatchers Feared. Note, 416
Open Letter to Whom it May Concern, An, . . . . . . . . . . . . . . . 27
*Organon*, Commentaries on the. B. Fincke, M. D., . . . . . . . . . 179
*Organon*, § 16. Arthur G. Allen, M. D., 430
*Organon*. The First Paragraph of. A Layman, . . . . . . . . . . . . 312
Our Dumb Animals. Notice of, . . . 528
Oxalis, . . . . . . . . . . . . . . . . 60

Palladium, . . . . . . . . . . . . . . 60
Paralysis, Infantile Spinal Atrophic. Thos. M. Dillingham, M. D., . . 564
Pasteur Institute, New York. Note. . 224
Patch, Frank W., M. D. The Treatment of the Uterine Displacements, . . . . . . . . . . . . . 188
Patents. Note, . . . . . . . . . . . . 80
Pease, F. O., M. D. Some Provings and Verifications, . . . . . . . 503
Pease, G. M., M. D. In Memoriam, . 218
Perverted Appetite and Impotence. C. M. Boger, M. D., . . . . . . . 116
Petroleum, . . . . . . . . . . . . . . 60
Phellandrium-aquat., . . . . . . . . 60
Philips, Dr. H. S. Removal of, . . . 464
Philosophic Relation of Homœopathy to Surgery, The. T. D. Stow, M. D., . . . . . . . . . . . . . . 488
Phosphorus, . . . . . . . . . . . . 93, 526
Physicians' Rebate Check-Book. By Wm. Jefferson Guernsey, M. D. Review of . . . . . . . . . . . 270
Physicians' Visiting List for 1892 and 1893. P. Blakiston, Son & Co. Notice of, . . . . . . . . . . . 32, 584
Phthisis Pulmonum. Some Cases of. Dr. Emil Schlegel, . . . . . . . 293
Physostigma, . . . . . . . . . . . . . 93
Phytolacca-decan., . . . . . . . 85, 93, 526
Pimpinella-sax., . . . . . . . . . . . 94
Pix liquida, . . . . . . . . . . . . . 94
Plantago-maj., . . . . . . . . . . . . 94

PAGE
Plantago-minor, . . . . . . . . . 94, 523, 527
Plumbum., . . . . . . . . . . . . . . . 85
Plumb-acet., . . . . . . . . . . . . . . 94
Pneumonia, . . . . . . . . . . . . . . 11
Podophyllum, . . . . . . . . . . . . . 86
Polygonum-hydrop., . . . . . . . . . 95
Polypi, Nasal—Cured with One Dose of Aurum-m. J. R. Haynes, M. D., . . . . . . . . . . . . . . 551
Pomeroy, Thos. F. In Memoriam, . 216
Postmaster-General, Annual Report of, . . . . . . . . . . . . . . . 76
Potassium Chlorate, Fragmentary Proving of. E. Rushmore, M. D., . . . . . . . . . . . . . . 530
Potentiation Physiologically Proven. Prof. Dr. G. Jaeger, . . 228, 328, 370
Potentization and Dose, Some Thoughts Upon. A. R. Morgan, M. D., . . . . . . . . . . . . . . 383
Practical Manual of Diseases of the Skin. By Geo. H. Rohé. Review of, . . . . . . . . . . . . 266
Pregnancy; A Case of Twins Simulating Fibroid Tumors. Leslie Martin, M. D., . . . . . . . . . . 149
Pre-Hahnemann Homœopathy. Note, 127
Prescriptions Based Upon Diagnosis. E. V. Ross, . . . . . . . . . . . 106
Primer of Materia Medica for Practitioners of Homœopathy, A. By Dr. Timothy Field Allen. Review of, . . . . . . . . . . . . 220
Proceedings of the I. H. A., The, . . 371
Provings and Clinical Observations with High Potencies. Malcolm McFarlan, M. D., . 50, 93, 134, 227, 522
Psoric Miasm, Thoughts on the. I. Dever, M. D., . . . . . . . . . . 456
Psorinum, . . . . . . . . . . . . 86, 95, 523
*Public Ledger* Almanac for 1892. Review of, . . . . . . . . . . . . . . 123
Pulex-irritans (Common Flea). W. A. Yingling, M. D., . . . . . . . . . 207
Pulsatilla . . . . . . . . . . . . . . . 96
Pye's Surgical Handicraft. By T. H. R. Crowle. Review of, . . . . . 410

Quackenbush, Dr. A. Note, . . . . . 317
Quackenbush, A., M. D. Laceration of the Cervix Uteri (?) Cured by Homœopathic Treatment, . . . 516

Ranunculus acris, . . . . . . . . . . 97
Ratanhia, . . . . . . . . . . . . . . 527
Reed & Carnrick's Preparations. Note, 271
Reflexes, The Doctrine of. Walter M. James, M. D., . . . . . . . . . . . 273
Repertories, The Use of. Note, . . . 62
Repertory, . . . . . . . . . . . . . . 86
Repertory to the Male Sexual Organs, A. Review of, . . . . . . . . 528
Resident Physicians Wanted. Note, . 127
Retina, Detachment of the. Clarence C. Howard, M. D., . . . . . . . 23
Rhododendron, . . . . . . . . . . . . 86, 97
Rhus-tox., . . . . . . . . . . . . 86, 97, 527
Rogers, Dr. Ida Wright. Note, . . . 414
Ross, E. V. Prescriptions Based upon Diagnosis, . . . . . . . . . . . 106
Rumex-crisp., . . . . . . . . . . . . 97
Rushmore, Dr. Edward. Note, . . . 125
Rushmore, E., M. D. Fragmentary Proving of Potassium Chlorate, 530
Sick Headache Cured with One Dose of Lachesis, . . . . . . . . . 64
Silica Symptoms Verified, . . . . 530
Ruta, . . . . . . . . . . . . . . . . 86

Sacch-lact., . . . . . . . . . . . . . 98
Sale. Not for. Walter M. James, M. D., 3
Salutatory Editorial. Walter M. James, M. D., . . . . . . . . . . 1
Salvia-off., . . . . . . . . . . . . . 99

PAGE
Sambucus-nig., . . . . . . . . . . . 99, 527
Sambucus in an Unusual Case of Sweating. J. C. White, M. D., . 115
Sanders, W. B. Note, . . . . . . . . . 79
Sanderson, P. C., M. D. Lachesis and Sepia Cases, . . . . . . . . . . 463
Syphilinum, . . . . . . . . . . . . 408
Sanguinaria, . . . . . . . . . . . . 99, 523
Santonine, . . . . . . . . . . . . . . 99
Sapo-soda, . . . . . . . . . . . . . 98, 523
(Sapo-cast), . . . . . . . . . . . . . . 522
Sarracenia-purp., . . . . . . . . . 100, 527
Sassafras, . . . . . . . . . . . . . . 100
Schlegel, Dr. Emil. Some Cases of Phthisis Pulmonum, . . . . . . 293
Sciatica, Notes on. B. Simmons, M. D., 83, 84
Scutellaria, . . . . . . . . . . . . . 134
Secale-cornutum, . . . . . . . . . . . 134
Selfridge, Dr. J. M. Note, . . . . . . 223
Selfridge, J. M., M. D. In Memoriam of Dr. Giles M. Pease, . . . . . . 260
Senega, . . . . . . . . . . . . . . 134, 523
Sepia, . . . . . . . . . . . . . . . 86, 134
Abscesses of Vulva Cured with. I. Dever, M. D., . . . . . . . . 548
Serpentaria, . . . . . . . . . . . . . 135
Sherbino, G. W., M. D Necrosis of Os Calcis, . . . . . . . . . . . . . 555
Should the Patient be Told What Remedy He Is Taking? Walter M. James, M. D., . . . . . . . . 225
Sick Headache, A Case of. Horace P. Holmes, M. D., . . . . . . . . . 37
Sides of the Body and Kindred Remedies. By Dr. C. von Bænninghausen. Review of, . . . . . . 463
Silica Symptom Verified. E. Rushmore, M. D., . . . . . . . . . . . 530
Silicea in the Treatment of Cancer. Note, . . . . . . . . . . . . . . 320
Silicea, . . . . . . . . . . . . . . . 135
Similia Similibus? Note, . . . . . 271
Similia a Universal Law? Is. J. R. Haynes, M. D., . . . . . . . . . . 448
Simmons, B., M. D. Notes on Sciatica, . . . . . . . . . . . . . . 83, 84
Simmons, S. S., M. D. A Proving of Oil of Wintergreen, . . . . . . . 462
Sir Arthur Sullivan. Note, . . . . . . 128
Skinner, Dr. Note, . . . . . . . . . 368
S. L. Some Remarks on Hamamelis Virginiana. Dr. Bree, . . . . . 61
Tanin Poisoning, . . . . . . . . . . 66
Tuberculinum Kochii, . . . . . . . 373
Smith, C. C., M. D. The Portrait of Dr. Adolph Lippe, . . . . . . . . 217
Smith, Sarah N., M. D. Concussion of the Brain, . . . . . . . . . . 400
Some Provings and Verifications. F. O. Pease, M. D., . . . . . . . . . 503
South, The. A Journal of Southern and Southwestern Progress. Review of, . . . . . . . . . . . 176
Specific Diagnosis. By John M. Scudder, M. D. Notice of, . . . . 77
Specific Medication. By John M. Scudder, M. D. Review of, . . 77
Spigelia, . . . . . . . . . . . . . . . 135
Spinal Congestion or Spinal Myelitis. Martin Leslie, M. D., . . . . . . 155
Spongia-tost, . . . . . . . . . . . . . 135
Stannum, . . . . . . . . . . . . . . . 136
Stanton, L. M., M. D. An Experience with Copper, . . . . . . . . . . 249
Stanton, L. M., Sec. The New York Homœopathic Union, . . . . . 3, 250
State and Municipal Boards of Health. Note, . . . . . . . . . . . . . . 128
Steinrauf, Wm., M. D. A Case of Metastasis, . . . . . . . . . . . . . 172
Cenchris Concortrix, . . . . . . 173
Hernia, . . . . . . . . . . . . . . 91
Stillingia-syl., . . . . . . . . . . . . 136

PAGE
Stow, T. Dwight, M. D. Fracture of the Femur. An Appeal, . . . . 297
The Philosophic Relation of Homœopathy to Surgery, . . . . . 488
Stramonium, . . . . . . . . . . . . . 86, 136
Strontia-carb., . . . . . . . . . . . . 135, 527
Strychnine Cure for Drunkenness, The, . . . . . . . . . . . . . . 35
Suggestions to Patients. By W. A. Yingling, M. D. Review of, . . 411
Sulph-ac., . . . . . . . . . . . . . 136, 523
Sulphur, . . . . . . . . . . . . . . 136
Surgical Cases, Three. Erastus Case, M. D., . . . . . . . . . . . . 543
Swan, S., M. D. Is it Homœopathy or Isopathy? . . . . . . . . . . . 75
Swan, Samuel, M. D. Key Notes, . . 164
Swan's Potencies, Dr. Note, . . . . 124
Swedenborg and Medicine. Chas. S. Mack, M. D., . . . . . . . . . . . 118
Sycosis, . . . . . . . . . . . . . . . 455
Sycosis Menti. Dr. Kunkel, . . . . . 200
Sycosis, Some Symptoms of. Geo. H. Clark, M. D., . . . . . . . . . . 451
Symphytum-off., . . . . . . . . . . . 137
Syphilis in Ancient and Prehistoric Times. By Dr. F. Buret. Review of, . . . . . . . . . . . . 409
Syphilinum. P. C. Sanderson, M. D., . 408

Tabacum, . . . . . . . . . . . . . . 137
Taft, Dr. Mary Florence, Removal of, 464
Tannin Poisoning. S. L., . . . . . . 66
Tapley, J. T. Nosode Cases, . . . . . 521
Tartar-emet., . . . . . . . . . . . . 138
Tellurium, . . . . . . . . . . . . . 86, 138
Temperature, A Phenomenal Case of High, . . . . . . . . . . . . . . 205
Terebinth, . . . . . . . . . . . . . 139, 524
Theridion-cuirass, . . . . . . . . . . 139
Think of it! Note, . . . . . . . . . 414
Thomas, Dr. Chas. M. Note, . . . . . 32
Thuja-occident., . . . . . . . . . . . 140
Thurston, Dr. Rufus Leander. Note, 414
Tonsillitis, . . . . . . . . . . . . . . 12
Transactions of the Fifteenth Annual Session of the California State Hom. Med. Soc. Review of, . . 366
Transactions of the Homœopathic Medical Society of the State of New York for the Year 1891. By John L. Moffat, M. D. Review of, . . . . . . . . . . . . . . . 268
Transactions of the Twenty-seventh Session of the Homœpathic Medical Society of Penna. Review of, . . . . . . . . . . . . 315
Transactions of the Twenty-seventh Annual Session of the Homœopathic Medical Society of State of Wisconsin. Review of, . . . 367
Traumatism, A Case of. Geo. A. Hermance, . . . . . . . . . . . . . . 107
Treat's International Medical Annual. Note, . . . . . . . . . . . . . . 78
Trifolium-album., . . . . . . . . . . 140
Trifolium-pract., . . . . . . . . . . . 140
Trigonocephalus-piscivorus, . . . . . 140
Trillium-pendulum, . . . . . . . . . 141
Triticum, . . . . . . . . . . . . . . 141
Trombidium, . . . . . . . . . . . . . 141
Tuberculinum Kochii. S. L., . . . . 373
Tussilago-farfara, . . . . . . . . . . 142
Tuttle, Ella M., M. D. A Symptom of Arsenic, . . . . . . . . . . . . . 29
Twenty-First Annual Report of Middletown State Hospital, New York, The. Notice of, . . . . . 123
Typhoid Fever Cured in Its First Stage. J. C. White, M. D., . . . 218

Use of Repertories in Finding the Homœopathic Remedy, The. Horace P. Holmes, M. D., . . . . . 36

PAGE
Utah Homœopathic Medical Association. Mrs. M. J. Green, M. D., . 309
Uterine Displacements, The Treatment of the. Frank W. Patch, M. D., . . . . . . . . . . . . . . 188
Uva-ursi, . . . . . . . . . . . . . 138, 527

Vaccination. Is the Act Compelling, Unconstitutional? Judge Thomas M. Wyatt, . . . . . . . . . . 478
Vaccination Is a Curse to the Human Race. C. A. Walters, M. D., . . 485
Vaccination, Discussion on, . . . . . . 482
Vaccination and Homœopathy. Harlyn Hitchcock, M. D., . . . . 476
Vaccination, Truth and Error in. Stuart Close, M. D., . . . . . . . 468
Vaccination. Truth and Error in. W. M. James, M. D., . . . . . . . . 465
Valerian, . . . . . . . . . . . . . . . 86
Valeriana-officinalis, . . . . . . . . 142
Van Baun, W. W., M. D. The Alumni Association of the Hahnemann Medical College, . . . . 173
Van Buren, Hattie C., M. D. Epilepsy—Dr. McLaren's Paper in March Number, . . . . . . . . . 265
Vandenburg, M. W., M. D. The Vital Force Defined, . . . . . . . . . 118
Variolinum, . . . . . . . . . . . . . 142
Verbascum-thapsus, . . . . . . . . . 142
Vick's Floral Guide, 1892. Notice of, 123
Villers, Alexander, M. D. Case Cured, 403
Vital Force Defined, The. Edward Bayard, M. D., . . . . . . . . . . 63
Vital Force Defined, The. M. W. Vandenburg, M. D., . . . . . . . . 118

Waggoner, G. J., M. D. A Case of Ulceration of the Lungs, . . . . . . 114
Walters, C. A., M. D. Is Vaccination a Curse to the Human Race? . . 485
Wanted. Note, . . . . . . . . . . . . 415
Weekly Bulletin, The, . . . . . . . . 317
Wells, L. B., M. D. The Oldest Homœopathic Physician, . . . . . . . . 113
Wells, P. P., M. D. In Memoriam—B. Fricke, M. D., . . . . . . . . . . 9
Wells on Intermittent Fever. Note, . 228
White, J. C., M. D. Coffea-cruda in Eneuresis, . . . . . . . . . . . 14
Stambucus in a Unusual Case of Sweating, . . . . . . . . . . . . . 115
Typhoid Fever Cured in its First Stage, . . . . . . . . . . . . . . 218
Why Their Bank Account Grows. Note, . . . . . . . . . . . . . . . 128
Wigg, George, M. D. Fearful Aggravation Caused by Laurocerasus, 30
Wildes, Thomas, M.D. Medorrhinum, 71
Cina, . . . . . . . . . . . . . . . 156
Winterburn, Dr. Geo. William. Note, 415
Wintergreen, A Proving of Oil of. S. S. Simmons, M. D., . . . . . . . . 462
Wisteria, . . . . . . . . . . . . . 142, 523
With the Pousse Café. By Wm. Tod Helmuth, M. D. Review of, . . 175
Wolff, M. A. A., M. D. In Memoriam, 8
Woman's Homœopathic Hospital, The, . . . . . . . . . . . . . . . 315
World's Columbian Exposition, The, 223
World's Congress Notes. Note, . . . 415
Worrari, . . . . . . . . . . . . . 143, 523
Wyatt, Judge Thomas. Is the Act Compelling Vaccination Unconstitutional? . . . . . . . . . . . 478

Yale Surgical Chair, The. Note, . . 80, 125, 317
Yingling, W. A., M. D. Pulex-irritans (Common Flea), . . . . . . . . . 207

Zinc-oxide, . . . . . . . . . . . . . . 143
Zinc-sulph, . . . . . . . . . . . . . . 143
Zingiber, . . . . . . . . . . . . . . . 143

VOL. XII. JANUARY, 1892. NO. 1.

418

### THE HOMŒOPATHIC PHYSICIAN,

A Monthly Journal of Homœopathic Materia Medica and Clinical Medicine.

*"He is a freeman whom truth makes free, and all are slaves beside."*

*"Seek the Truth: come whence it may, cost what it will."*

EDITED AND PUBLISHED BY

WALTER M. JAMES, M. D.

NOTICE.

***All articles for publication, books for review, business communications, etc., must be sent to Editor, 1125 Spruce Street, Philadelphia.***

***Subscribers will please notice that this Journal will be sent until arrears are paid and it is ordered discontinued.***

PHILADELPHIA:

No. 1125 SPRUCE STREET.

